# Long‐term nutrient addition increases respiration and nitrous oxide emissions in a New England salt marsh

**DOI:** 10.1002/ece3.3955

**Published:** 2018-04-20

**Authors:** Rose M. Martin, Cathleen Wigand, Elizabeth Elmstrom, Javier Lloret, Ivan Valiela

**Affiliations:** ^1^ ORISE Postdoctoral Participant Narragansett RI USA; ^2^ EPA Atlantic Ecology Division Narragansett RI USA; ^3^ The Ecosystems Center Marine Biological Laboratory Woods Hole MA USA; ^4^ University of Washington Seattle WA USA

**Keywords:** carbon dioxide, cavity ringdown spectroscopy, Great Sippewissett Marsh, methane, nitrous oxide, nutrient enrichment

## Abstract

Salt marshes may act either as greenhouse gas (GHG) sources or sinks depending on hydrological conditions, vegetation communities, and nutrient availability. In recent decades, eutrophication has emerged as a major driver of change in salt marsh ecosystems. An ongoing fertilization experiment at the Great Sippewissett Marsh (Cape Cod, USA) allows for observation of the results of over four decades of nutrient addition. Here, nutrient enrichment stimulated changes to vegetation communities that, over time, have resulted in increased elevation of the marsh platform. In this study, we measured fluxes of carbon dioxide (CO_2_), methane (CH_4_) and nitrous oxide (N_2_O) in dominant vegetation zones along elevation gradients of chronically fertilized (1,572 kg N ha^−1^ year^−1^) and unfertilized (12 kg N ha^−1^ year^−1^) experimental plots at Great Sippewissett Marsh. Flux measurements were performed using darkened chambers to focus on community respiration and excluded photosynthetic CO_2_ uptake. We hypothesized that N‐replete conditions in fertilized plots would result in larger N_2_O emissions relative to control plots and that higher elevations caused by nutrient enrichment would support increased CO_2_ and N_2_O and decreased CH_4_ emissions due to the potential for more oxygen diffusion into sediment. Patterns of GHG emission supported our hypotheses. Fertilized plots were substantially larger sources of N_2_O and had higher community respiration rates relative to control plots, due to large emissions of these GHGs at higher elevations. While CH_4_ emissions displayed a negative relationship with elevation, they were generally small across elevation gradients and nutrient enrichment treatments. Our results demonstrate that at decadal scales, vegetation community shifts and associated elevation changes driven by chronic eutrophication affect GHG emission from salt marshes. Results demonstrate the necessity of long‐term fertilization experiments to understand impacts of eutrophication on ecosystem function and have implications for how chronic eutrophication may impact the role that salt marshes play in sequestering C and N.

## 1 INTRODUCTION

In salt marshes, complex, interacting biogeochemical processes drive greenhouse gas (GHG) fluxes. As coastal waters become increasingly eutrophic worldwide (Bricker, Clement, Pirhalla, Orlando, & Farrow, 1999; Bricker et al., 2008; Kirwan & Megonigal, 2013; Paerl, Hall, Peierls, & Rossignol, 2014; Rabalais, Turner, Díaz, & Justić, 2009; Valiela, 2015), nutrient‐stimulated changes to salt marsh structure and function may alter GHG fluxes and therefore salt marshes’ role in global climate (Mcleod et al., 2011).

Nutrient enrichment alters salt marsh plant community composition (Rogers, Harris, & Valiela, 1998). The elevation constraints of most salt marsh plant species, and the tight interaction of species with sediment conditions, including redox and relative elevation, generate a series of interactions. Plant species present on marsh swards occur in well‐constrained bands determined to a large degree by submergence regime (Bertness, 1991), but marsh plants release O_2_ and oxidizing compounds to sediment (Lovell, 2005). Plant taxa in marshes hence both depend upon and affect redox within sediments, and effects vary with relative elevation in the tidal frame.

Studies have demonstrated the effects of over four decades of fertilization (Valiela, 2015) on plant communities and elevation at Great Sippewissett Marsh in Cape Cod, MA, USA (Fox, Valiela, & Kinney, 2012; Rogers et al., 1998; Valiela, 2015). Large decadal‐scale changes within vegetated plots subject to high nutrient enrichment resulted in increased *S. alterniflora* height and biomass, as well as changes in species cover, with *Distichlis spicata* becoming more dominant (Valiela, 2015). Changes in elevation and subsequent feedbacks on vegetation communities likely affect associated rhizosphere microbial processes and soil biogeochemistry, and C and N cycles that drive GHG fluxes.

When not subjected to eutrophic conditions, salt marshes are characterized by high rates of C sequestration and relatively small GHG emissions (Mcleod et al., 2011). Salt marshes are productive ecosystems with waterlogged sediments, largely anaerobic, that support heterotrophic metabolism dominated by fermentative and sulfate reducing bacteria (Howarth & Teal, 1979). Methane production is limited by dominance of sulfate reduction pathways (Bartlett, Bartlett, Harriss, & Sebacher, 1987; Poffenbarger, Needelman, & Megonigal, 2011). Reported CH_4_ emissions at Great Sippewissett Marsh are small (11–34 μmol m^−2^ hr^−1^) (Howes, Dacey, & Teal, 1985) and comparable to other salt marshes in the region (Martin & Moseman‐Valtierra, 2015).

Salt marsh CO_2_ production includes plant root and shoot and sediment microbial respiration. Great Sippewissett Marsh sediment CO_2_ emissions have been previously reported (10–13 mmol m^−2^ s^−1^) (Howes et al., 1985). Carbon dioxide produced in salt marsh soils is frequently exceeded by photosynthetic CO_2_ uptake during the growing season (Martin & Moseman‐Valtierra, 2015, 2016, 2017a; Moseman‐Valtierra et al., 2016), and emissions are small during cooler months (Martin & Moseman‐Valtierra, 2015).

Nitrous oxide production in salt marsh sediment may result from microbial processes including denitrification and nitrification (Kool et al., 2010; Koop‐Jakobsen & Giblin, 2010; Wrage, Velthof, van Beusichem, & Oenema, 2001). Nitrous oxide production is dependent on oxygen availability in sediments: under anaerobic conditions, nitrate undergoes complete denitrification to N_2_ gas. In salt marshes not subjected to eutrophication, N is limiting (Valiela & Teal, 1974) and so N_2_O fluxes generally are small. N_2_O emissions were found to be negligible at high and low marsh elevations of several southern New England salt (Martin & Moseman‐Valtierra, 2015; Moseman‐Valtierra et al., 2011) and brackish (Martin & Moseman‐Valtierra, 2015, 2017a) marshes receiving low N loads.

Nutrient enrichment drives changes in salt marsh structure and function via a number of mechanisms and may ultimately increase GHG emissions. Nutrient enrichment accelerates salt marsh litter decomposition and CO_**2**_ emission (Deegan et al., 2012; Wigand, Brennan, Stolt, Holt, & Ryba, 2009). Where changes in vegetation communities have driven elevation increases, less saturated sediments may support increased aerobic metabolism and greater CO_2_ emission due to respiration. Coupling of oxic and anoxic conditions where soil is less saturated may support biogeochemical pathways that, in combination with N‐replete conditions, result in N_2_O emission. Therefore, elevation gains may facilitate N_2_O production by incomplete denitrification (Koop‐Jakobsen & Giblin, 2010) and nitrifier denitrification (Kool et al., 2010; Wrage et al., 2001) and are likely also suboptimal for N_2_O uptake resulting from denitrification (Chapuis‐Lardy et al., 2007). Increased N_2_O emission with N enrichment has been demonstrated by mesocosm (Martin & Moseman‐Valtierra, 2017b), field nutrient pulse (Moseman‐Valtierra et al., 2011), and 6‐year enrichment (Chmura, Kellman, van Ardenne, & Guntenspergen, 2016) experiments. Chronic enrichment, such as that characteristic of coastal waters worldwide (Rabalais et al., 2009), could produce a sustained N_2_O emission response (Murray, Erler, & Eyre, 2015), potentially increasing radiative forcing of eutrophic salt marshes as N_2_O has 263 times the global warming potential of CO_2_ (Neubauer & Megonigal, 2015).

There is a need for long‐term experiments to test effects of chronic nutrient enrichment on salt marsh GHG fluxes. In this experiment, we tested effects of ~45 years of nutrient enrichment (using an organic NPK fertilizer containing multiple N species) at Great Sippewissett Marsh on CO_2,_ CH_4_ and N_2_O fluxes measured with opaque chambers in situ. We related GHG fluxes in experimental plots to vegetation communities and elevation. As N retention in the Great Sippewissett plots is high and exports are minor (<7%) (Brin, Valiela, Goehringer, & Howes, 2010), this experiment allowed relative certainty of N loading rates to our experimental plots. We hypothesized that (1) N enrichment will stimulate N_2_O emission, especially at higher elevations where soil saturation is decreased, coupling oxic, and anoxic conditions that drive higher ratios of N_2_O/N_2_ emissions from denitrification; (2) CO_2_ emissions will be greater due to increased community respiration in the nutrient enriched treatment and at higher elevations where decreased sediment saturation and increased O_2_ availability support more rapid decomposition, and (3) CH_4_ emissions will vary with elevation, with smallest emissions at higher elevations where sediment O_2_ availability is greatest.

## 2 MATERIALS AND METHODS

### 2.1 Site description and experimental design

**Figure 1:**
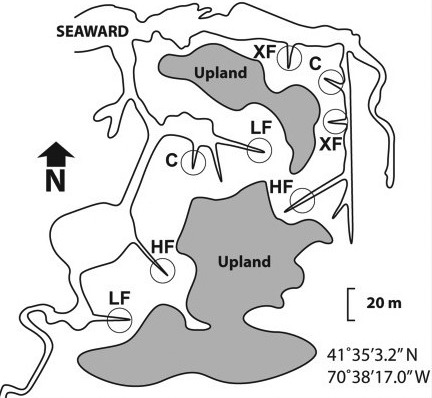
Experimental plot locations in Great Sippewissett Marsh. This study focused on XF (fertilized; 1,572 kg N  ha^−1^ year^−1^) and C (Control; 12 kg N  ha^−1^ year^−1^) plots (labeled to match previous publications on the experimental plots)

This study was conducted at plots experimentally nutrient‐enriched for over 45 years (and continuing) in Great Sippewissett marsh, Cape Cod (Valiela, 2015) (Figure 1). Organic fertilizer is hand‐broadcast every 2 weeks, March‐October, each year, with treatments that include an unfertilized control and three doses of nitrogen (N) using Milorganite, a commercially available mixed NPK fertilizer (10% N, 6% P, 4% K by weight) containing ammonium nitrate, potassium nitrate, ammonium sulfate, and urea, among other constituents (http://www.milorganite.com).

For this study, we focused on GHG fluxes from two replicate control (C) plots (no fertilization, 12 kg N ha^−1^ year^−1^ from external sources) and two replicate plots that received high doses of N (XF) (1,572 kg N ha^−1^ year^−1^). We measured GHG fluxes monthly from July‐October 2016 at three elevation zones (Creekbank, Mid and High) in XF plots and 2 (Creekbank and Low) in C plots (Table 1), with six or eight replicate plots per zone. Due to distinct topography responses to nutrient addition, high and medium elevation zones have no counterpart in C plots, and the low elevation zone has no counterpart in XF plots. Elevation and vegetation in XF plots display striking responses to four decades of fertilization, as previous studies have described in detail (Fox et al., 2012; Rogers et al., 1998). In XF plots, relative elevations ranged from 0 to −69 cm relative to MHW. Tall *S. alterniflora* dominates along the creekbank, *D. spicata,* and intermediate ht. *S. alterniflora* dominate at Mid elevations, and *I. frutescens* dominates high elevations (Table 1). In C plots, the elevation range is much narrower (from −36 to −70 cm relative to MHW). Short *S. alterniflora* dominates at low elevations and tall *S. alterniflora* dominates creek banks (Table 1).

**Table 1:** Average relative elevations, percent cover, and dominant plant species of elevation classes for C and XF treatments

	Elevation class			
	Creekbank	Low	Mid	High
Relative elevation (cm)^a^				
C	−62.5 ± 2.4	−41.2 ± 1.5		
XF	−59.2 ± 3.4		−24.9 ± 0.7	−5.5 ± 1.8
Percent cover of elevation class within plot (%)^b^				
C	14.8 ± 10.1	83.6 ± 6.3		
XF	12.7 ± 1.3		54.5 ± 11.3	36.0 ± 16.1
Dominant plant species				
	Tall *Spartina alterniflora*	Short *Spartina alterniflora*	Intermediate *Spartina alterniflora*,* Distichlis spicata*	*Iva frutescens*,* Atriplex patula*

a Elevations relative to mean high water. Averages (8–14 points per elevation class).

b Percent cover of elevation zones estimated using dominant plant species and the Braun‐Blanquet method. Average ± *SE* of replicate plots (*n* = 2) are shown.

### 2.2 Relative elevation and vegetation measurements

We measured relative elevation within vegetation zones using wooden laths (1 m height; *n* = 6 or 8 per vegetation zone, within 0.5 m of flux measurement locations) which were painted with a mix of food dye and water‐soluble glue and deployed just before high tide (Smith & Warren, 2012). We recorded heights of watermarks to determine elevation of vegetation zones relative to mean high water (Table 1). We estimated percent cover of each elevation zone within each of the XF and C plots using established vegetation cover survey methods for the dominant species in each defined elevation zone (Braun‐Blanquet, 1932).

### 2.3 Greenhouse gas flux measurement

To define the effect of elevation, vegetation, and nutrient enrichment on GHG fluxes, we designed 0.3 m diameter × 0.6 m tall cylindrical static flux chambers to encompass vegetation without damage. Chambers sit snugly in PVC collars installed about 6 cm into the marsh surface, and we achieved a gas‐tight seal by adding a small amount of water to the gap between collar and chamber. To prevent water pooling or shading effects within collars, we installed collars approximately 1 hr prior to gas flux measurements. We repeated measurements at the same points, marked with small flags, during each visit. We performed flux measurements at *n* = 6 or eight locations for Creekbank, low and high elevation classes. For the Mid elevation classes, where the dominant species (*D. spicata* and *S. . alterniflora*) formed patches, we increased replication to *n* = 12–16. We used a cavity ring down spectroscopy (CRDS) in situ analyzer (Picarro G2508) to measure CO_2_, CH_4_, and N_2_O concentrations in real time (Martin & Moseman‐Valtierra, 2015). We covered chambers with white, light‐blocking fabric to exclude photosynthetic CO_2_ uptake and measured community respiration and GHG gas fluxes. We conducted all GHG flux measurements for 4–6 min per plot (with approximately one‐second sampling intervals), based on observed periods for linear rates of change and to avoid excessive chamber warming (Brannon et al., 2016; Martin & Moseman‐Valtierra, 2015). To record air temperature at 30‐s interval, we suspended Hobo^®^ data loggers (Onset, Bourne, MA, USA) within the chamber during all flux measurements. We used the Ideal Gas Law to calculate changes in gas concentrations using within‐chamber air temperatures, ambient atmospheric pressure, and chamber volume and footprint area. Using published, empirically determined minimum detectable slopes of concentration over time for each gas for the Picarro analyzer (Brannon et al., 2016), we computed minimum detectable fluxes for this experiment accounting for chamber size and measurement durations: 0.07 μmol m^−2 ^hr^−1^ (CH_4_); 0.44 μmol m^−2 ^hr^−1^ (N_2_O) and 0.0001 mmol m^−2 ^hr^−1^ (CO_2_). When the *p*‐value of the comparison of the slope with a horizontal line was >0.01 (indicating no concentration change), we assigned fluxes a value of zero.

### 2.4 Statistical analyses

We used linear mixed effects models for analysis of the effect of vegetation zone on CH_4_, N_2_O, and CO_2_ fluxes from each treatment type (XF and C plots). We treated vegetation type as a fixed effect and sampling month and plot as random effects. To test for effects of treatment (XF or C) for Tall *S. alterniflora* (present in plots of both treatments), we used linear mixed effects models with treatment as the fixed effect and sampling month and plot as random effects. To obtain *p*‐values to assess significance of the effect of vegetation zone and treatment on GHG fluxes, we performed likelihood ratio tests of full models against models with the fixed vegetation zone or treatment effect removed. We performed all statistics in R (Team, 2013) and interpreted significance at α = 0.05.

## 3 RESULTS

### 3.1 N_2_O fluxes

**Figure 2:**
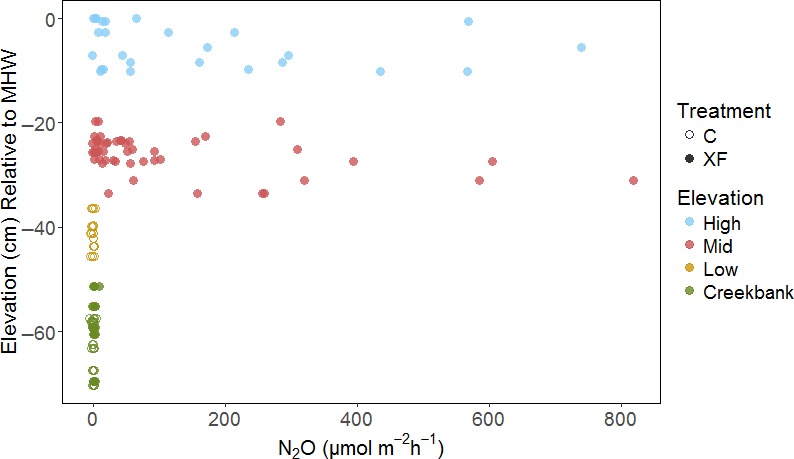
Scatter plot of N_2_O fluxes collected throughout the experiment and elevation relative to mean high water (MHW). N_2_O fluxes from C and XF plots are represented by open and closed circles, respectively. Elevation classes are represented by different colors. Mid and high elevation classes are present only in XF plots; the low elevation class is present only in C plots, and the Creekbank zone is present in both XF and C plots

**Figure 3:**
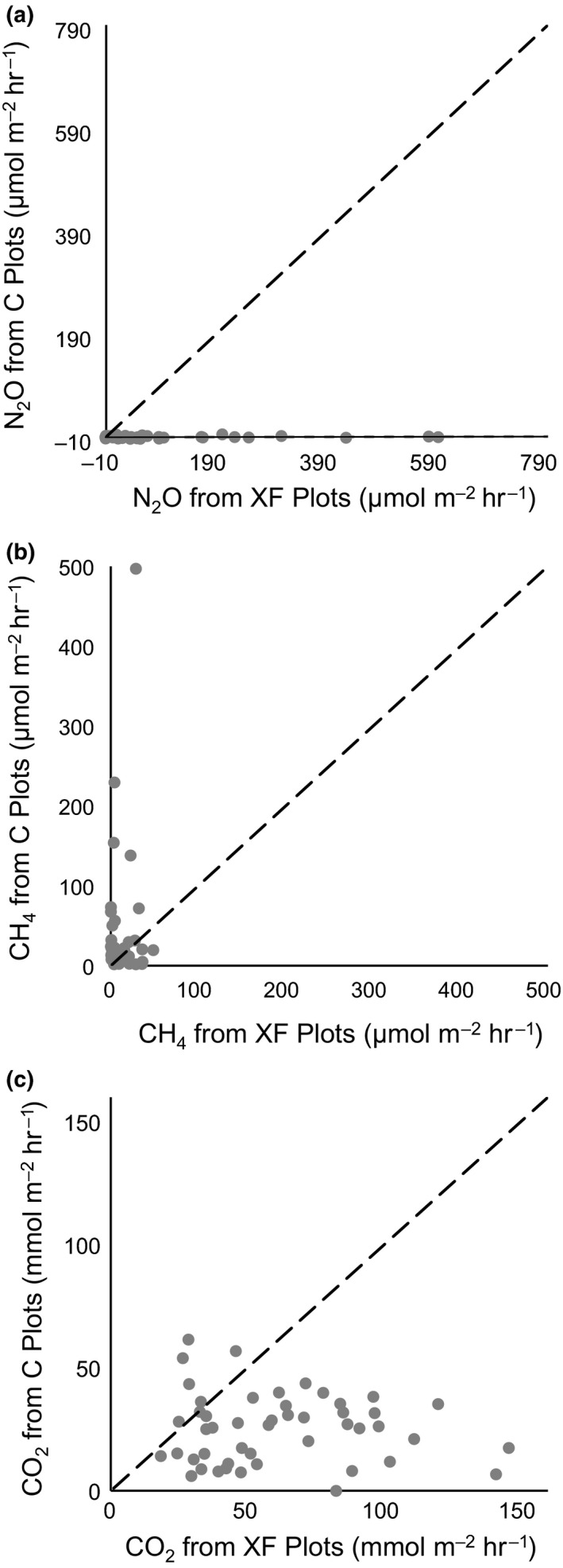
Scatter plots of N_2_O (a), CH_4_ (b), and CO_2_ (c) fluxes from C vs. XF plots. Dashed lines represent a 1:1 relationship between XF and C plot fluxes. Lines of best fit for the scatter plots are shown. Deviance of plots from a 1:1 relationship represents differences in fluxes between C and XF plots

Detectable N_2_O emissions were restricted to XF plots at medium and high elevation zones (relative elevation to MHW > −25 cm) (Table 2, Figure 2). This finding is consistent with the hypothesis that less saturated sediment and N‐replete conditions may favor microbial transformations that produce N_2_O. In C plots, N_2_O emissions were minimal, with the majority of fluxes below detection of the Picarro G2508 analyzer (Table 2, Figure 3a). N_2_O fluxes in the XF plots differed significantly between vegetation zones (Table 3), with large emissions in the Mid and High zones, and negligible emissions in Creekbank zone. Emissions in the Creekbank zone were below detection in both XF and C plots.

**Table 2:** Average greenhouse gas fluxes (±*SE*) measured monthly from C and XF plot elevation classes

	Elevation class			
	Creekbank	Low	Mid	High
XF Plots				
N_2_O flux (μmol m^−2 ^hr^−1^)				
July	0.0 ± 0.0^a^		37.2 ± 8.0	283.2 ± 75.6
August	2.1 ± 1.6		187.9 ± 70.4	14.4 ± 8.0
September	1.0 ± 0.7		53.0 ± 29.0	64.7 ± 27.0
October	−0.2 ± 0.2^a^		136.2 ± 60.8	271.1 ± 142.6
CH_4_ flux (μmol m^−2 ^hr^−1^)				
July	29.2 ± 5.4		15.2 ± 3.7	0.9 ± 0.4
August	27.9 ± 2.6		6.7 ± 1.6	0.2 ± 0.2^a^
September	53.1 ± 15.5		6.0 ± 1.3	0.2 ± 0.2^a^
October	8.0 ± 19		1.9 ± 0.6	0.1 ± 0.1^a^
CO_2_ flux (mmol m^−2 ^hr^−1^)				
July	36.1 ± 4.8		82.1 ± 9.7	73.6 ± 15.4
August	39.1 ± 4.8		72.4 ± 8.6	31.5 ± 3.9
September	29.6 ± 3.3		49.1 ± 5.5	35.0 ± 9.9
October	8.5 ± 1.5		28.2 ± 3.8	63.5 ± 16.0
C Plots				
N_2_O flux (μmol m^−2 ^hr^−1^)				
July	−0.5 ± 0.3	0.0 ± 0.7^a^		
August	−1.4 ± 0.7	−1.8 ± 0.9		
September	0.2 ± 0.4^a^	−0.9 ± 0.9		
October	−0.2 ± 0.2^a^	0.0 ± 0.0^a^		
CH_4_ flux (μmol m^−2 ^hr^−1^)				
July	16.5 ± 3.4	34.4 ± 13.4		
August	37.2 ± 11.8	129.3 ± 84.7		
September	14.2 ± 3.1	85.5 ± 41.0		
October	11.2 ± 4.9	19.5 ± 9.9		
CO_2_ flux (mmol m^−2 ^hr^−1^)				
July	28.6 ± 6.6	30.3 ± 3.0		
August	39.6 ± 6.7	37.4 ± 2.1		
September	20.3 ± 3.2	28.5 ± 2.9		
October	12.4 ± 3.2	9.0 ± 0.7		

*n *=* *6 to 8 for Creekbank, low and high elevation classes, and 12–16 for the mid elevation class.

a Below instrument detection limit.

**Table 3:** Results of linear mixed effects model tests for differences among vegetation zones and post hoc Tukey's HSD tests for greenhouse gas fluxes

	Effect of vegetation zone	Tukey's HSD test results
**XF Plots**		
N_2_O flux	$\chi_{3}^{2}$ = 13.1, *p* = .001^a^	Creekbank^a^
		Mid^b^
		High^b^
CH_4_ flux	$\chi_{3}^{2}$ = 68.1, *p* < .001^a^	Creekbank^a^
		Mid^b^
		High^c^
CO_2_ flux	$\chi_{3}^{2}$ = 19.6, *p* < .001^a^	Creekbank^a^
		Mid^b^
		High^b^
**C Plots**		
CH_4_ fluxes	$\chi_{1}^{2}$ = 3.4, *p* = .06	
CO_2_ fluxes	$\chi_{1}^{2}$ = 1.5, *p* = .22	

χ^2^ statistics, degrees of freedom, and *p* values are shown for likelihood ratio tests comparing null and full models for effect of vegetation zone on GHG fluxes. In Tukey's HSD columns, vegetation zones not sharing the same letter are significantly different.

Net GHG fluxes are computed using global warming potentials of 34 for CH_4_ and 300 for N_2_O. No detectable N_2_O fluxes were measured in the control (C) treatment.

a Significant at α = 0.05.

### 3.2 CH_4_ Fluxes

Methane emissions varied with elevation, with negligible emissions at high elevations (Table 2). While CH_4_ emissions differed significantly between elevation zones in XF plots, short and Creekbank *S. alterniflora* zones in C plots had similar emissions (Table 3). Creekbank CH_4_ emissions did not differ between XF and C plots, demonstrating that nutrient enrichment did not affect CH_4_ emissions in this zone. Since elevations are lower in the C plots, generally more CH_4_ was produced in C than XF plots (Table 2). One very large flux (1,351.8 μmol m^−2^ hr^−1^), seemingly due to ebullition based on the “stepwise” pattern of increase evident from plots of CH_4_ concentration over time (Middelburg et al., 1996), was measured in the short *S. alterniflora* zone in a C plot in August, signifying that short‐term measurements may not capture ebullition events and therefore may underestimate total CH_4_ emissions.

### 3.3 CO_2_ Fluxes

Carbon dioxide emission (respiration) was greatest in XF plots at the mid and high elevations (Tables 2, 3). Results support the hypothesized greater respiration rates at higher elevations, although Creekbank *S. alterniflora* emissions did not differ between XF and C plots, demonstrating that fertilization did not directly affect CO_2_ emission in this zone. As higher elevations emitted more CO_2_, XF plots generally produced more CO_2_ due to respiration than C plots (Figure 3c). As was expected as measurements were performed using darkened chambers to exclude photosynthetic uptake, CO_2_ emission was observed during all measurements.

## 4 DISCUSSION

**Figure 4:**
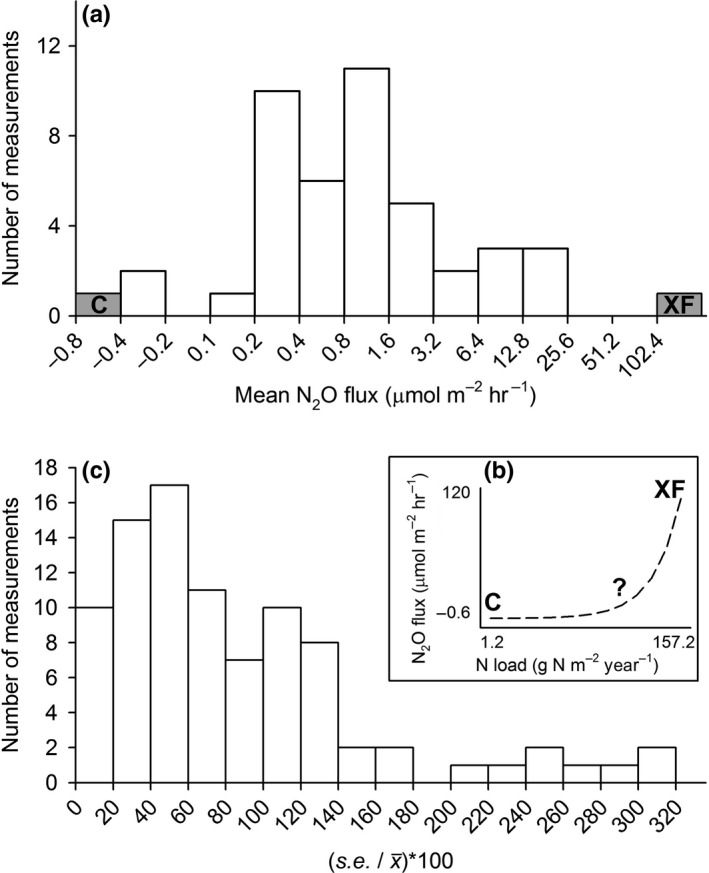
(a) Histogram showing mean N_2_O fluxes from salt marshes compiled in Murray et al. (2015) (white), and measured in control (C) and fertilized (XF) experimental plots at Great Sippewissett (grey); (b) Data on N_2_O fluxes in Great Sippewissett C and XF plots shown vs. the experimental N inputs; (c) Variability of measurements (as standard error expressed as a % of the mean) of N_2_O fluxes from salt marshes, from compilation in Murray et al. (2015)

The nutrient enrichment experiments at Great Sippewissett Marsh represent, to our knowledge, the longest‐running experiment of its kind from which GHG fluxes have been measured. While salt marsh CO_2_ and CH_4_ fluxes have received considerable attention, N_2_O fluxes remain poorly constrained with reported ranges varying widely (Murray et al., 2015) (Figure 4). Our reported N_2_O emissions are larger than the majority of those that have been reported previously (Figure 4). Given widespread recent eutrophication of coastal waters (Rabalais et al., 2009), our results suggest the potential for salt marshes to increasingly serve as N_2_O sources.

While some patterns were clear, our findings are limited by the Picarro analyzer's detection limit for N_2_O. Our results suggest a relative elevation threshold (−25 cm MHW) above which N_2_O emissions increase substantially with nutrient enrichment (Figure 2). Fluxes at lower elevations may have varied between treatments or among elevation zones but been below the detection limit of the analyzer (0.44 μmol m^−2 ^hr^−1^) (Brannon et al., 2016). Detecting small emissions is important as, given the large global warming potential of this potent GHG, small increases in emission per unit area could have an outsized impact on salt marsh radiative forcing. To refine the apparent elevation threshold for N_2_O, future investigations should employ techniques, such as use of available analyzers with lower detection limits (Brannon et al., 2016), to test for finer‐scale differences in N_2_O emissions from low marsh elevations. Future investigations could make use of the long‐term nutrient loading gradient at Great Sippewissett Marsh, and associated changes in vegetation communities and elevation, to discern finer‐scale responses of N_2_O emissions to nutrient loading. Examinations of edaphic conditions such as pore water nutrient availability and soil Eh across elevation gradients in these plots would help to clarify mechanisms driving observed patterns of N_2_O emissions.

Results clearly demonstrate larger CO_2_ emissions due to community respiration from higher‐elevation vegetation zones in XF plots as hypothesized, but there is a need for further investigation to determine whether increased productivity due to nutrient enrichment drives increased photosynthetic CO_2_ uptake in this system. A number of studies report measurements of negative net ecosystem exchange (NEE), the net CO_2_ flux resulting from plant photosynthesis and community respiration (performed using transparent flux chambers), for southern New England salt marshes. For example, in highly productive tall *S. alterniflora* (Moseman‐Valtierra et al., 2011) and *Phragmites australis* (Martin & Moseman‐Valtierra, 2015, 2016, 2017a,b) marshes, photosynthetic CO_2_ uptake exceeded respiration rates during the growing season, resulting in substantial net GHG uptake (up to 54 mmol m^−2^ hr^−1^). However, NEE measures indicated CO_2_ emission or minimal uptake in less productive *S. patens* and *D. spicata* marshes (Martin & Moseman‐Valtierra, 2015).

Nutrient addition has been shown to increase salt marsh aboveground biomass production (Adam Langley, Mozdzer, Shepard, Hagerty, & Patrick Megonigal, 2013; Deegan et al., 2012; Valiela, Teal, & Sass, 1975), and therefore, both community respiration and photosynthetic CO_2_ uptake may increase. At Great Sippewissett Marsh, in the early years of the fertilization experiment, *S. alterniflora* biomass increased (Valiela et al., 1975), and greater *S. alterniflora* productivity was associated with higher root zone Eh in a positive feedback loop (Howes et al., 1981). Both photosynthetic CO_2_ uptake and CO_2_ produced by microbial metabolism are likely increased in XF relative to C plots. However, shifts in species composition may complicate productivity responses to fertilization (Langley & Megonigal, 2010). There is in particular a need for data on effects of nutrient enrichment on salt marsh vegetation communities dominated by deeper‐rooting woody species including *Iva frutescens*, which may influence soil processes through rhizosphere processes such as ventilation and water uptake.

Although elevation gains mediated trade‐offs in GHG emissions by supporting decreased CH_4_ emission as well as increased CO_2_ and N_2_O emission, CH_4_ emissions in this system ranged from negligible to small relative to those from freshwater wetlands (Mitsch & Gosselink, 2000). Results of this study and others in similar systems (Martin & Moseman‐Valtierra, 2015; Moseman‐Valtierra et al., 2011) underline the relatively minor role of CH_4_ in salt marsh GHG emissions (Poffenbarger et al., 2011).

This experiment demonstrated the potentially significant impact that chronic nutrient enrichment may have on the salt marsh GHG emissions, particularly those of N_2_O. Nitrogen loading rates for the XF plots at Great Sippewissett Marsh receive (1,572 kg N ha^−1^ year^−1^), placing it within range of eutrophic Northeastern US salt marshes (Wigand, McKinney, Charpentier, Chintala, & Thursby, 2003; Wigand et al., 2014). Nutrient enrichment is a widespread stressor of salt marsh ecosystems, as eutrophication of coastal waters continues to increase globally (Bricker et al., 1999; Rabalais et al., 2009). The potential for nutrient enrichment to increase GHG emissions has implications for the role that salt marshes play in sequestering C and thereby countering climate change, an ecosystem service for which they are often valued (Mcleod et al., 2011). However, other studies that have tested effects of nutrient enrichment and used different N forms and application methods have found different marsh structural responses (subsidence of creekbanks and loss of structural integrity) (Deegan et al., 2012). Therefore, to improve understanding of nutrient enrichment impacts on salt marsh GHG emissions, there is a need for future studies to test GHG flux responses to structural changes in long‐term fertilization experiments employing varying N forms, loading rates, and application methods.

In conclusion, the shift in plant communities and subsequent gains in elevation driven by decades of nutrient enrichment at Great Sippewissett Marsh led to larger N_2_O emissions and higher community respiration rates. Future experiments to determine the proportion of these GHG emissions that may be offset by gains in productivity and to discern biogeochemical mechanisms underlying GHG emissions are needed, as are GHG flux measurements across a variety of nutrient enrichment experiments employing different N forms and delivery mechanisms. Nevertheless, results of this experiment have implications for the salt marsh ecosystem functions of C and N transformation and sequestration. Results highlight the need for long‐term experiments, such as the fertilization study at Great Sippewissett Marsh, to test effects of nutrient enrichment on salt marsh ecosystem structure and functioning.

## CONFLICT OF INTEREST

None declared.

## AUTHOR CONTRIBUTIONS

Rose M. Martin, Ph.D. designed experiment, collected and analyzed data, main author of manuscript. Cathleen Wigand, Ph.D. and Elizabeth Elmstrom contributed to experimental design, collected data, reviewed manuscript. Javier Lloret provided Figure 4 and contributed to the manuscript discussion. Ivan Valiela, Ph.D. provided extensive site background and expertise, contributed to experimental design, contributed to manuscript.
